# Pregnancy outcomes among SARS-CoV-2-infected pregnant women with and without underlying diseases: a case-control study

**DOI:** 10.25122/jml-2021-0157

**Published:** 2021

**Authors:** Samereh Ghelichkhani, Ensiyeh Jenabi, Ebrahim Jalili, Azam Alishirzad, Fatemeh Shahbazi

**Affiliations:** 1. Deputy of Treatment, Hamadan University of Medical Sciences, Hamadan, Iran; 2. Autism Spectrum Disorders Research Center, Hamadan University of Medical Sciences, Hamadan, Iran; 3. Department of Emergency Medicine, School of Medicine Besat Hospital, Hamadan University of Medical Sciences, Hamadan, Iran; 4. School of Nursing and Midwifery, Hamadan University of Medical Sciences, Hamadan, Iran; 5. Department of Epidemiology & Biostatistics, School of Public Health, Hamadan University of Medical Sciences, Hamadan, Iran

**Keywords:** comorbidity, pregnancy outcomes, coronavirus, pregnancy

## Abstract

This study aimed to examine the pregnancy outcomes in women infected with the severe acute respiratory syndrome coronavirus 2 (SARS-CoV-2) with and without underlying diseases in western Iran. This case-control study compared 49 pregnant women with Coronavirus disease (COVID-19) with underlying diseases (the case group) and 49 women with COVID-19 without underlying diseases (the control group). The groups were pregnant women with COVID-19 admitted to Hamadan hospitals for delivery. COVID-19 was diagnosed by using the reverse transcriptase-polymerase chain reaction (real-time RT-PCR). Data were evaluated using a checklist. Further, the Statistical Package for the Social Sciences (SPSS) version 16 was used for data analysis. A value of p<0.05 was considered statistically significant. The odds of preterm labor were five times higher in women with underlying diseases (OR=5.95, 95% CI (3.01, 7.15), p=0.034). Moreover, the odds of preeclampsia and eclampsia in women with underlying diseases was (OR=3.35, 95% CI (1.18, 4.93), p=0.048) and (OR=2.65, 95% CI (1.43, 3.54), p=0.035), respectively. The results revealed that preterm labor, preeclampsia, and eclampsia were significantly higher in women with COVID-19 and underlying diseases compared to those without underlying diseases. Thus, the need to identify and educate pregnant mothers on underlying diseases and attention to prenatal care, particularly in high-risk groups, is necessary for the COVID-19 pandemic.

## Introduction

Acute Respiratory Syndrome Coronavirus 2 (SARS-CoV-2) is a virus causing Coronavirus disease (COVID-19). Information on COVID-19 is rapidly progressing, and temporary guidance from different organizations is constantly updated and expanded [1]. Emerging infections can have a significant effect on pregnant women and their fetuses [2]. Limited information is available on COVID-19 during pregnancy. Nonetheless, information on other highly pathogenic coronavirus-associated diseases, such as Severe Acute Respiratory Syndrome (SARS) and Middle East Respiratory Syndrome (MERS), may provide insight into the effects of the disease during pregnancy.

The main symptoms of this disease range from fever and cough to severe respiratory illness and death [2, 3]. Although pregnancy does not seem to increase susceptibility to infection, most COVID-19 mothers recover. However, pregnant women are at greater risk for a severe illness, increasing the need for maternal intensive care and mechanical ventilation [4].

Some researchers have shown pregnant women with risk symptoms, such as age over 35, obesity, and hypertensive and diabetic signs, had more severe form of COVID-19 [5–7]. Which of the following underlying diseases in pregnant women with COVID-19 causes worse pregnancy outcomes is a key question, the answer to which could be useful in resolving the significant challenges countries face in maintaining maternal and neonatal health care services during pregnancy, as well as during and after delivery. As no studies have been performed in Iran in this regard, the present research compares risk factors and pregnancy outcomes among pregnant women with COVID-19 with and without underlying diseases.

## Material and Methods

The present case-control study was performed among women referred for delivery to hospitals of the Hamadan province located in the West of Iran from March 2020 until April 2021. During this period, 49 women with underlying diseases were referred to hospitals of the Hamadan province for delivery.

The case and control groups were SARS-Cov-2-infected pregnant women referred for delivery to hospitals of the Hamadan province. The COVID-19 disease was diagnosed via real-time reverse-transcriptase polymerase-chain-reaction (real-time RT-PCR) using nasal and nasopharyngeal specimens as samples. The cases were women with at least one underlying disease such as heart conditions, diabetes, autoimmune diseases, chronic respiratory conditions, cancer, or asthma. The control group had no underlying disease.

For each SARS-Cov-2-infected pregnant woman with underlying diseases, one SARS-Cov-2-infected woman without underlying diseases was selected. A total of 49 women were included in each group. Further, the groups were matched based on their place of residence.

Data were collected by a checklist including information on maternal age, gestational age, education level, occupation, gravidity, parity, preterm labor (defined as gestational age of less than 37 completed weeks), preeclampsia, eclampsia, delivery type, low birth weight [LBW], fetal distress, meconium stain, maternal mortality, and neonate death. The validity and reliability of the checklist were assessed in advance.

Characteristics of the SARS-Cov-2 positive pregnant women cases were presented as number (%) for categorized variables and mean (SD) for continuous variables for symptomatic and asymptomatic women. The Shapiro-Wilk test was used to check the normality distribution of the investigated variables. Chi-square was used to compare background characteristics between the two groups. Also, the comparison of continuous variables between two groups was carried out by the independent t-test. Logistic regression was used to explain the relationship between the study group and pregnancy conditions and delivery outcomes. For the data analysis, the Statistical Package for the Social Sciences (SPSS) version 16 was used. A p-value of ≤0.05 was considered statistically significant.

## Results

A total of 49 pregnant women contributed to each of the case and control groups infected with SARS-Cov-2. The baseline characteristics of patients in the groups are compared in Table 1.

**Table 1.** Characteristics of the case and control groups.

**Characteristics**	**Cases (n=49) n (%)**	**Controls (n=49) n (%)**	**P-value**
**Age**			
<20 20–30 31–40 >40	3 (6.12) 19 (37.77) 26 (53.06) 1 (3.05)	1 (2.04) 22 (44.90) 23 (46.94) 3 (6.12)	0.352
**Education**			
Elementary Guidance High School University	11 (22.45) 17 (34.69) 14 (28.57) 7 (14.29)	10 (20.41) 18 (36.73) 18 (36.73) 3 (6.13)	0.461
**Occupation**			
Housewife Employee	28 (57.14) 21 (42.86)	34 (69.39) 15 (30.61)	0.286
**Gravidity**			
1 2 3 ≥4	23 (46.94) 13 (26.53) 9 (18.37) 4 (8.16)	16 (32.65) 19 (38.77) 12 (24.49) 2 (4.09)	0.203
**Parity**			
0 1 2 3	24 (48.99) 13 (26.53) 10 (20.41) 2 (4.07)	17 (34.69) 19 (38.77) 10 (20.41) 3 (6.13)	0.489
**Number of abortions**			
0 1 2	42 (85.71) 5 (10.20) 2 (4.09)	40 (81.63) 9 (18.37) 0 (0.00)	0.160
**Gestational age (weeks)**			
<32 32–34 35–36 37–42	6 (12.24) 21 (42.86) 4 (8.17) 18 (36.73)	5 (10.20) 5 (10.20) 1 (2.04) 38 (77.56)	0.015
**Type of delivery**			
Normal vaginal delivery Cesarean delivery	19 (38.77) 30 (61.23)	27 (55.10) 22 (44.90)	0.218

The two groups – case and control – were homogenous concerning maternal age, gestational age, delivery type, number of abortions, education, occupation, parity, and gravity (p>0.05), while gestational age in the two groups was statistically significant (p=0.015).

In Table 2, we compared pregnancy and delivery outcomes between the two groups. The odds of preterm labor in women with underlying diseases were five-fold higher (OR=5.95, 95% CI (3.01, 7.15), p=0.034). Moreover, the odds of preeclampsia and eclampsia were significantly higher in women with underlying diseases (OR=3.35, 95% CI (1.18, 4.93), p=0.048) and (OR=2.65, 95% CI (1.43, 3.54), p=0.035), respectively.

**Table 2.** Comparison of pregnancy and delivery outcomes in the two groups.

**Variable**	**Women with underlying diseases (n=49)**	**Women without underlying diseases (n=49)**	OR (95%CI)*	**P-Value**
**Preterm labor, n (%)**	31 (63.26)	11 (22.45)	5.95 (3.01, 7.15)	0.034
**Preeclampsia, n (%)**	19 (38.77)	8 (16.33)	3.35 (1.18, 4.93)	0.048
**Eclampsia, n (%)**	15 (32.56)	7 (14.28)	2.65 (1.43, 3.54)	0.035
**Cesarean delivery, n (%)**	30 (60.46)	19 (38.77)	1.00 (0.73, 2.18)	0.064
**Low birth weight (LBW), n (%)**	30 (62.79)	23 (46.94)	1.78 (0.96, 2.55)	0.053
**Death rate in neonates, n (%)**	2 (4.08)	0 (0.00)	-	-
**Maternal mortality, n (%)**	3 (6.12)	0 (0.00)	-	-
**Fetal distress, n (%)**	10 (20.41)	9 (18.37)	1.14 (0.96, 1.73)	0.096
**Meconium stain, n (%)**	3 (6.12)	0 (0.00)	-	-

* women without underlying diseases were considered as the reference group

Although cesarean section rates (60. 46% vs. 38.77%) and LBW (62.79% vs. 46.94) were higher in women with underlying diseases, these differences were not statistically significant (p>0.05). The present study showed 3 (6.12%) maternal deaths and 2 (4.08%) neonate deaths in the case group. In addition, 3 (6.12%) meconium stain was reported in the case group.

## Discussion

Our results revealed that the odds of preterm labor, preeclampsia, and eclampsia were significantly increased in SARS-Cov-2-infected women with underlying diseases compared to those without underlying diseases.

A similar study conducted in Mexico showed that underlying diseases, such as hypertension, diabetes, and obesity, were not only related to the severity of the disease induced by SARS-Cov-2 but also provided the base for COVID-19 [8]. Kaim *et al.* indicated an increasing incidence of severe consequences in mothers with a history of hypertension and preeclampsia with coronary artery disease [9]. The study results revealed a five-fold increase in the odds of preterm labor in women with COVID-19 and underlying diseases. The odds of developing preeclampsia and eclampsia among the women with underlying diseases increased significantly in the study. These results are consistent with those of Schwartz *et al.*, based on which SARS-Cov-2-infected pregnant women with underlying diseases are more likely to develop preeclampsia, gestational hypertension, uterine scarring, gestational diabetes, and uterine atony [10].

Lokken *et al.* reported that pregnant women hospitalized for a SARS-CoV-2 concern were more likely to have comorbidities or underlying conditions such as asthma, hypertension, type 2 diabetes mellitus, autoimmune disease, and morbid obesity [11]. Delahoy *et al.* showed that 20.6% of pregnant women hospitalized with COVID-19 had at least one underlying disease, including asthma (8.2%) and hypertension (4.3%) [12].

In Iran, Janabi *et al.* indicated that the odds of cesarean delivery and low birth weight infants in women with COVID-19 symptoms significantly increased compared to women without the disease [13]. Although the cesarean section rate was 60.46% vs. 38.77% and LBW 62.79% vs. 46.94% in women with underlying diseases versus women without underlying diseases, respectively, these differences found in this study were statistically insignificant (p<0.05). The study showed 3 cases (6.12%) of maternal deaths and 2 cases (4.8%) of neonatal deaths in the case group.

However, there were some limitations of this study. As the sample size was limited, all potential confounders could not be controlled. It is recommended that future studies use a larger sample size. Another limitation was the lack of follow-up of mothers and infants in the postpartum period. Thus, it is recommended to investigate the maternal and neonatal results in the postpartum period.

## Conclusion

According to the study results, the odds of preterm labor, preeclampsia, and eclampsia in SARS-Cov-2-infected women with underlying diseases were significantly higher than those without underlying diseases. Thus, it is necessary to take measures to enhance maternal care management in health centers with close monitoring of pregnant mothers’ conditions suspected or affected in terms of pregnancy risk symptoms by being aware of the potential for harm among pregnant women who are in the second or third trimester and suffer from underlying diseases and COVID-19.

## Acknowledgments

### Ethical approval

The ethical approval for this study was obtained by the authors from the Ethics Committee of the Hamadan University of Medical Sciences (approval ID: IR.UMSHA.REC.1400.001).

### Data availability

The datasets used are available from the corresponding author upon reasonable request.

### Consent to participate

Written informed consent was obtained from the participants.

### Conflict of interest

The authors declare that there is no conflict of interest.
